# Role of NADPH Oxidases as Novel Therapeutic Targets for the Impaired Neurovascular Unit in the Early Stage of Diabetic Retinopathy

**DOI:** 10.3390/ijms27041879

**Published:** 2026-02-15

**Authors:** Stavroula Dionysopoulou, Kyriaki Thermos

**Affiliations:** Department of Pharmacology, School of Medicine, University of Crete, 70013 Heraklion, Greece; stav_dionisopoulou@yahoo.com

**Keywords:** diabetic retinopathy, neurovascular unit, neurodegeneration, vasculopathy, neuroinflammation, NADPH oxidases, NOX inhibitors, neuroprotection

## Abstract

Diabetic Retinopathy is the most common microvascular complication of diabetes. The Neurovascular Unit (NVU) is brought to the surface for its importance to retinal physiological function. Diabetes impairs the NVU through diverse causative factors, such as ischemia, oxidative stress, and excitotoxicity. The interplay between members of the above triad leads to the main pathological factors of Diabetic Retinopathy—namely, neurodegeneration, neuroinflammation, and vasculopathy. Emphasis is given to the pathology of the early stage of Diabetic Retinopathy (ESDR) and the putative new therapeutic treatments that will prevent/delay the development of the advanced stage of the disease, in which vision is compromised. NADPH oxidases (NOX1-NOX5), whose main function is to produce reactive oxygen species and induce oxidative/nitrative stress will be presented as novel therapeutic targets for the impaired Neurovascular Unit. The knowledge of the molecular mechanisms involved in the neuroprotection induced by novel specific inhibitors of NOX2 and NOX4 against the diabetic insults will confer the hope that therapeutic treatments for ESDR will evolve in the near future and be beneficial to millions of subjects who are in the early stage of Diabetic Retinopathy, as well as subjects with other complications of diabetes.

## 1. Introduction

Diabetes mellitus is a chronic, metabolic disorder which is rapidly affecting individuals around the world. Data from the International Diabetes Federation (IDF) indicate that in 2024, 588.7 million people globally had been diagnosed with diabetes, while this number is expected to rise to 852.5 million by 2050 [1]. The detrimental impact of chronic hyperglycemia to blood vessel physiology and integrity leads to the development of macrovascular and microvascular complications in multiple organs [2].

Diabetic Retinopathy (DR)is the most common microvascular complication of diabetes and the leading cause of preventable blindness in the working age population in developed countries [3,4,5]. It has been estimated that in 2020, 103.12 million people were living with DR, 28.54 million people suffered from vision-threatening Diabetic Retinopathy (VTDR), and 18.83 million were diagnosed with diabetic macular edema (DME). These numbers are expected to rise by 2045 to 160.50, 44.82, and 28.61 million for DR, VTDR, and DME, respectively [6].

Chronic exposure to hyperglycemia and other causal factors, such as hypertension and lipid abnormalities, is believed to initiate a cascade of biochemical and physiological alterations that ultimately lead to microvascular damage and retinal dysfunction. The clinical hallmarks of PDR include increased vascular permeability and DME due to the breakdown of the blood–retinal barrier (BRB), which causes vascular microaneurysms and endothelial cell proliferation, all of which lead to neovascularization. Due to these pathological conditions, DR was originally considered a microangiopathy, an exclusively micro vascular disease [4].

Increasing evidence has suggested that neurodegeneration is also involved in the pathophysiology of DR. Investigations in subjects [7,8] and animal models of DR [9,10,11] reported that neurodegeneration is an early event in the timeline of DR and it may also precede microvascular complications. Neurodegeneration underlies the mechanisms leading to retinal cell death, suboptimal visual acuity and blindness [4,12,13]. The hyperglycemia-induced alterations in various biochemical pathways lead to oxidative damage of micro-vessels in retina. The pathways involved are the formation of advanced glycation end products (AGE) and the activation of its receptor, RAGE, activation of hexosamine, polyol, and protein kinase C (PKC) [14].

Based on fundus detection of these microvascular lesions, DR was categorized as non-proliferativeDiabetic Retinopathy (NPDR), which is characterized by microaneurysms, hemorrhages, hard exudates [15], and PDR, in which excessive ischemia triggers an abnormal neovascularization response. The International Council of Ophthalmology also published a classification of DR, employed by clinicians, dependent on the recognition of specific anatomical disturbances. This classification further separates NPDR into mild, moderate and severe [16]. More recent clinical observations pertaining to the symptoms observed in the different stages of DR, along with preclinical studies in animal models of the disease, contributed to the development of a novel system for DR classification, placed in the early and advanced stage of Diabetic Retinopathy, ESDR and ASDR, respectively.

ASDR includes severe NPDR and PDR [13], also known as VTDR [6,17] and DME. No visible lesions were noted under the traditional fundoscopic examination of patients in the ESDR, but there was evidence consistent with early neuro-retinal deterioration [18,19] and small-scale microvascular complications [20]. Thus, ESDR includes a phase without symptoms, mild and moderate NPDR. The timely identification (screening) of preclinical DR is essential in order for the clinician to select, (a) subjects with diabetes who are at a greater risk for developing ASDR, and (b) the most effective management of the disease.

The therapeutic treatment for ESDR is presently based solely on the control of risk factors, such as blood glucose, dyslipidemia, and hypertension. Insulin, fibrates (fenofibrate), and antihypertensive treatment have a significant effect on subjects with type 2 diabetes [21]. However, some subjects with diabetes with tight glycemic and blood pressure control still develop DR [22]. The FIELD [23] and ACCORD STUDY [24] concluded that fenofibrate, a peroxisome proliferator activated–receptor alpha (PPAR) agonist, a treatment for dyslipidemia, reduced the risk of development and progression of DR in subjects with type2 diabetes. However, other investigations reported that fenofibrate mediates, via other mechanisms, anti-apoptotic activity, and improvement of endothelial function, prevention of BRB breakdown, anti-angiogenic activity, antioxidant and anti-inflammatory events [25,26,27,28].

Current pharmacological treatments focus on the ASDR [29], the stage in which vision has been compromised. Antivascular endothelial growth factor (anti-VEGF) agents, administered intravitreally, treat only the microangiopathy/vasculopathy, whereas corticosteroids reduce inflammation. However, the positive and promising results observed with the anti-VEGF treatments are hampered by their adverse side effects, such as cataract formation, retinal detachment, vitreous hemorrhage and potential loss of neural retinal cells [30]. Most importantly, they do not cause total regression of neovascularization, and do not reverse vision loss. Other commonly used treatments include laser photocoagulation, and invasive vitreo-retinal surgical procedures [31,32].

The asymptomatic nature of the initial phase of ESDR, in combination with the lack of effective treatments, may be a major cause for increasing the prevalence of ASDR. These put an enormous burden on the healthcare systems and impair the quality of life of subjects with diabetes [33]. The recognition of the pathologies of ESDR is very important for the development of new therapeutic treatments for the postponement of the ASDR and blindness.

## 2. Neurovascular Unit and Diabetes

Hyperglycemia induces the dysregulation of different metabolic pathways [34]. Chronic exposure to hyperglycemia, along with other factors, such as hypertension and lipid abnormalities, mentioned earlier, initiates a cascade of events that lead to the dysregulation of metabolic pathways and production of causative factors, such as ischemia, oxidative stress, and excitotoxicity [35,36]. These causative factors and the interplay between them lead to neurodegeneration, inflammation, and vasculopathy.

The American Diabetes Association (ADA) has defined DR as a highly tissue-specific neurovascular complication [37]. The term “Neurovascular Unit” (NVU) was first described in brain but was also introduced in retina. NVU describes the complex interactions, interdependency, and functional coupling of neurons, glia cells (macroglia/microglia), and the components of the vasculature (endothelial cells, pericytes) [4].

The different neural cell types [retinal ganglion cells (RGCs), bipolar, horizontal, and amacrine cells], together with glia cells [macroglia: Müller cells, astrocytes, and microglia] constitute the “neural part” of the NVU, while endothelial cells and pericytes compose the “vascular part”. Each specific cell type of NVU has a distinct role. Their coordinated function and close communication ensure the maintenance of the inner blood–retinal barrier (iBRB), as well as the regulation of blood flow locally, depending on the metabolic status of neural retina. Communication between the different cell types is achieved either directly, through physical interactions (e.g., endothelial cells and pericytes) in a paracrine and/or autocrine manner [38,39,40,41,42].

Oxidative stress (OS), excitotoxicity, imbalance in neurotrophic factors, glial dysfunction, and apoptotic cell death occur in diabetes and lead to severe dysregulation and disruption of the NVU homeostasis, causing BRB breakdown, structural and functional impairments on neurons and the vasculature [31,38,39,43]. The activation of macro- and microglia and the release of pro-inflammatory cytokines introduce a hyperglycemia-induced inflammatory component [44], which provides the link between the neural and vascular impairment of the NVU [45]. Neurodegeneration, inflammation, and vasculopathy appear to be responsible for the impairment of the NVU and BRB disruption, both critical for the pathogenesis of the ESDR. It has been reported that retinal dysfunction is present in subjects with diabetes lacking microvascular abnormalities. These latter findings led to the suggestion of renaming the microangiopathy disease, Diabetic Retinopathy (DR) to Diabetic Retinal Disease (DRD), a name in which both neurodegenerative and microvascular pathologies are involved [37,46].

New knowledge regarding the role of neurodegeneration and the impairment of NVU in the development of DR has led to a better understanding of the pathogenesis of the early stage of the disease [40]. In addition, the development of animal models of ESDR to assess the pharmacological profile of new therapeutic strategies targeting both neurodegeneration and microvascular abnormalities in diabetic animals has been established. The streptozotocin (STZ) model of DR in rodents has been employed in a large number of investigations, in which the therapeutic agents under study, namely, peptides [47,48,49], microneurotrophins [50,51], endocannabinoids [52], NADPH oxidase inhibitors [53,54] and others, are administered topically, as eye drops or intravitreally in different chronological (length of treatment) protocols. The db/db mice were also employed as a useful model for the study of neuroprotection against retinal neurodegeneration, by different pharmacological agents [55,56]. The findings of the above-mentioned investigations suggested that ESDR models and routes of administration of putative therapeutics under study protected the diabetic retina against neurodegeneration and vasculopathy. Most importantly, it has been determined that topical administration, as eyedrops, of diverse therapeutic targets, is an effective route of administration with many advantages, including the limitation of systemic side effects, the avoidance of invasive methods, and subject friendly treatment [57]. This is particularly important when using small molecules because some of them can reach the retina at pharmacological concentrations very rapidly by the trans-scleral route [58,59].

**Figure 1:**
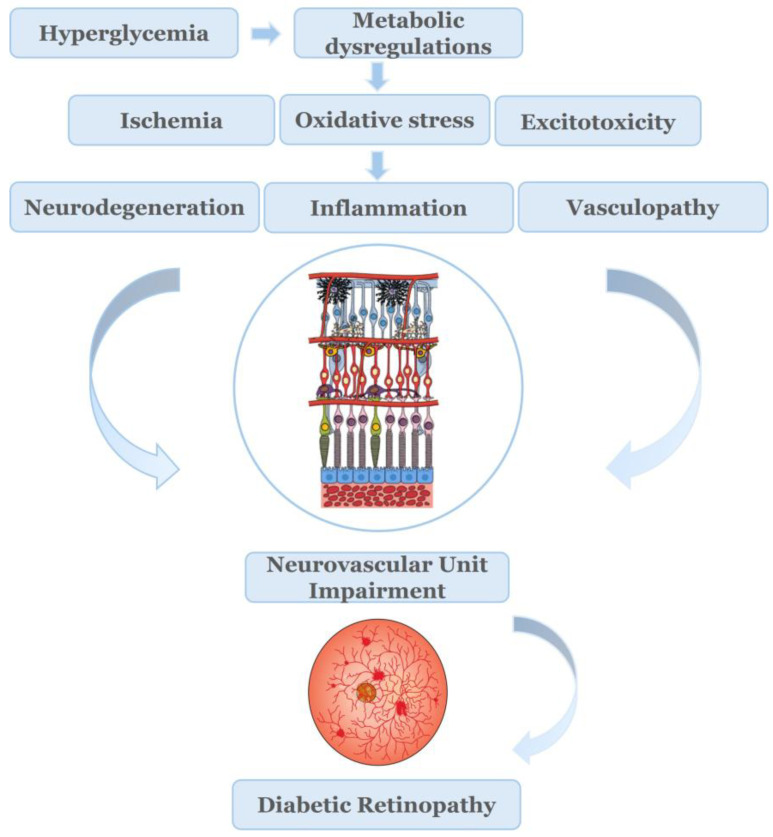
Mechanisms leading to Neurovascular Unit impairment in Diabetic Retinopathy.Chronic hyperglycemia, through the dysregulation of various metabolic pathways, can cause the manifestation of ischemic and excitotoxic conditions in retina, and the development of oxidative stress. These causative factors render the retinal tissue vulnerable to inflammatory insults, neurodegenerative and vascular changes, leading to the severe impairment of the retinal NVU and subsequently to the development of Diabetic Retinopathy [35,36,37].

OS emerges as an important causative factor in the development of DR implicated in the impairment of NVU. NOX represent a major source of reactive oxygen species (ROS) in retina that is responsible for the development OS (Figure 1). This review focuses on the role of NADPH oxidases (NOX1, NOX2, NOX4) in the early stage of DR, their cross talk with OS and excitotoxicity, and as targets for the development of novel therapeutics for the pathologies instigated by the impairment of the NVU.

## 3. NADPH Oxidases (NOX): Physiology and Function

**Figure 2:**
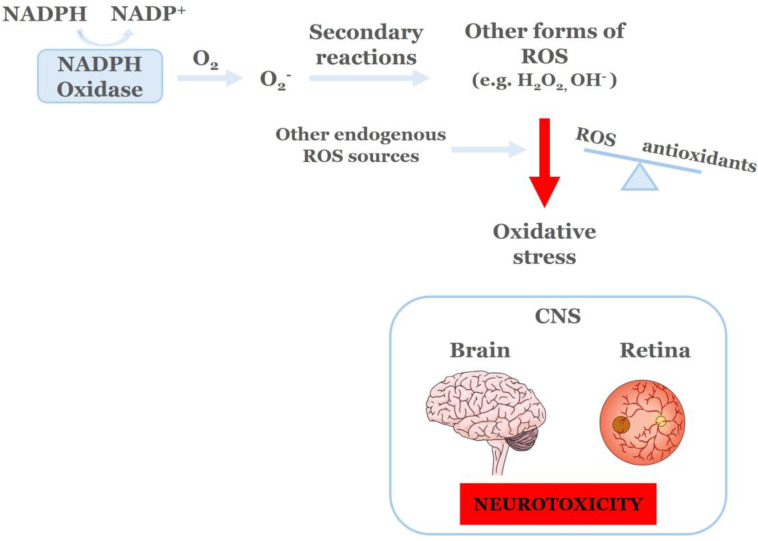
NADPH Oxidases (NOX) function and oxidative stress.NADPH oxidases’ sole role is the production of reactive oxygen species (ROS, O_2_^−^, H_2_O_2_). Different pathological stimuli, such as DR, cause an imbalance between ROS and antioxidant factors, due to increased production of ROS, from NOX and other endogenous ROS generating systems, leading to the development of oxidative stress. ROS; Reactive Oxygen Species, CNS; Central Nervous System [61].

NADPH oxidases (NOX) area family of enzymes that include seven known isoforms, namely NOX1-NOX5, DUOX1/2 [60]. The main role of NOX is to produce (ROS) by catalyzing the transfer of one electron from NADPH to molecular oxygen, leading to the production of superoxide (O_2_^−^) (Figure 2). The newly formed O_2_^−^, through secondary reactions, will lead to the production of other forms of ROS, including hydrogen peroxide (H_2_O_2_) and hydroxyl radicals (OH·). Normally, the NOX-derived ROS, as well as those produced from other endogenous sources (mitochondria, xanthine oxidase, cytochrome P450 oxygenases, lipoxygenase and cyclooxygenase), are essential for a plethora of molecular and cellular processes, including host defense and the regulation of gene expression and cell signaling [61]. NOX isoforms are major sources of cytosolic ROS [62].

**NOX1 isoform**: It represents the first identified homolog of NOX2 [63]. The NOX1 enzymatic complex includes the trans membrane subunits NOX1 and p22phox, along with the cytosolic subunits NOX organizer 1 (NOXO1) [61]. It also requires the recruitment of Rac1 regulatory protein for the activation of the complex and the subsequent generation of superoxide [64,65].

**NOX2 isoform**: It was the prototypic enzyme of the NADPH oxidase family. NOX2 consists of two membrane subunits, gp91phox and p22phox, that form the NOX2 core, known as cytochrome b558, and three cytosolic subunits, p47phox, p67phox, and p40phox. Upon activation, phosphorylation of p47phox leads to the interaction with the p22phox membrane subunit and the subsequent recruitment of the cytosolic subunit sp67phox and p40phox to the NOX2 core. Rac1 or Rac2 regulatory protein binds directly to p67phox, resulting in the formation of the NOX2 activated enzymatic complex that catalyzes the production of superoxide [61,66,67].

**NOX4 isoform**: Unlike the other NOX isoforms, it is constitutively active. It exhibits a unique ROS generation pattern, with its rapid conversion of superoxide to hydrogen peroxide [68]. The NOX4 dependent production of ROS also requires interaction with the membrane subunit p22phox, but the function of the NOX4 isoform seems to be independent of other cytosolic regulatory proteins, including the Rac1 GTPase [69]. Interaction between p22phox and NOX4 in the endoplasmic reticulum suggested a unique mechanism of NADPH oxidase complex formation [70]. The interaction of the regulatory protein poldip2 with p22phox has been correlated with increased NOX4 enzymatic activity and its constitutive character [71], something that has to be further investigated. Most recently, NOX3 expression and function in retinal ganglion cells and amacrine cells was reported [72].

### 3.1. NADPH Oxidases as Critical Regulators of Oxidative Stress

OS refers to the imbalance between the excess production of ROS and their reduction from the endogenous antioxidant systems, thus leading to severe cell damage and death. Its source is from the metabolic abnormalities induced by hyperglycemia [61], but it also enhances these abnormalities causing a lethal result.

In the Central Nervous System [CNS (brain and retina)], OS leads to neurotoxicity and has been identified as one of the major mechanisms underlying the pathogenesis of vasculopathy, neurodegeneration and neuroinflammation. As mentioned above, the two major sources of ROS are NADPH oxidases (cytosolic) and mitochondria (oxidative phosphorylation, electron transport chain) [61,73].

Most cell types in CNS express multiple NOX isoforms that are differentially regulated, have distinct sub-cellular localizations and serve unique roles. Apart from their roles in the normal physiology of brain and retina, NOX isoforms are correlated with diverse CNS pathologies. In brain, NOX4, located in endothelial cells and neurons, is linked to the breakdown of the BRB and neuronal cell death that leads to ischemic stroke [74]. In retina, NOX1, NOX2, and NOX4 are expressed in RGCs, amacrine and endothelial cells, pericytes, and macro- and microglia. Their activation under pathological conditions leads to vascular dysfunction and neurodegeneration. Many investigations also reported the role of NOX in the development of inflammatory conditions [75,76,77,78].

Abnormal activity of transcription factors, such, as nuclear factor-κB (NF-κB) [79], hyperglycemia-mediated mitochondrial damage [80,81], the reduced activity of the transcription factor, nuclear factor erythroid-2-related factor 2 (Nrf2) and epigenetic regulation [82,83,84] and others [85], are correlated with ROS and DR. Even though many investigations have been performed to ascertain the exact molecular mechanisms involved in the role oxidative stress in the development of DR, further research is required to provide conclusive answers.

### 3.2. NADPH Oxidases and Retinal Ischemia

Ischemia is the underlying cause of many ocular diseases, including DR that leads to retinal cell loss, neovascularization, and blindness. Retinal ischemia induces the release of excess glutamate, from retinal photoreceptors, bipolar, and ganglion cells. Glutamate is the leading neurotransmitter in retina. It initiates a cascade of events that lead to excitotoxicity via the activation of different receptor subtypes belonging to ionotropic, NMDA (N-methyl-D-aspartate), AMPA (RS)-α-amino-3-hydroxy 5-methyl-4-isoxazole propionic acid hydrobromide, kainate and metabotropic receptors [86,87].Glutamate activation of NMDA receptors leads to excessive increase in calcium ions (Ca^2+^), activation of nitric oxide synthetase, and the release of nitric oxide (NO), thus, an increase in reactive oxygen and nitrative stress (ROS/RNS), leading to neuronal cell death. NMDA excitotoxicity has been used extensively as a model for retinal neurodegenerative disease, since NMDA receptors are located on RGCs, whose axons are responsible for forming optic nerves. NMDA excitotoxicity leads to RGC death and visual loss [88];thus, it may be considered a model of the ASDR.

Besides the Ca^2+^ permeable NMDA receptor, activation of other ionotropic glutamatergic receptors, as mentioned above, the non-NMDA receptors, AMPA and kainate, are also involved in retinal excitotoxicity. Their use as experimental models has played an important role in the evaluation of ischemia-related cell death and neuroprotection in retinal disease. The toxic effects of AMPA induced the attenuation of the number of viable retinal cells, activated macroglia (Müller cells) and microglia [89,90,91]. AMPA receptors are composed of tetrameric subunits, GluA1–4 that form homomeric or heteromeric complexes that are differentially expressed in retinal neurons [92]. AMPA receptors lacking the GluA2 subunit are permeable to Ca^2+^ and their activation leads to excitotoxicity.

AMPA excitotoxicity in experimental animals reduced retinal cell viability of cholinergic, and nitric oxide synthetase (NOS)-expressing amacrine cells, and horizontal cells, but had no effect on photoreceptors, bipolar or ganglion cells [93]. These findings led to the conclusion that AMPA excitotoxicity is an effective model for the study of the early events of retinal ischemia, and in the assessment of the neuroprotective properties of new pharmacological agents. Other studies from our lab [94,95,96] suggested that AMPA excitotoxicity not only leads to neurodegeneration, but also to neuroinflammation. The above constitute the molecular mechanisms involved in the excitotoxicity induced retinal neurodegeneration and neuroinflammation.

We also reported for the first time in the literature that AMPA increased ROS levels and OS in rodent retina. This increase was mediated by NADPH oxidases. The latter was confirmed by the use of specific inhibitors for NOX1, NOX2 and NOX4, all three displaying differential patterns, regarding their role as putative neuroprotectants and anti-inflammatory agents [97]. The findings presented in this Section 3.2 propose that there is interplay between excitotoxicity, NOX and OS in retina leading to neurodegeneration and neuroinflammation.

## 4. NADPH Oxidase Involvement in the Development of Diabetic Retinopathy

Studies using different in vitro and in vivo models of DR have correlated hyperglycemia with increased expression and enzymatic activity of NADPH oxidases, including NOX1/4 [75,98], NOX2 [54,99] and NOX4 [53,78] isoforms present in retinal cells. Moreover, in humans, a genome-wide association study has linked the NOX4 gene with increased risk of severe DR in subjects with diabetes type 2 diabetes, suggesting the involvement of NADPH oxidases in diabetes-induced retinal abnormalities [100]. An important mechanism that is associated with the upregulation of NOX isoforms in DR is the hyperglycemia induced activation of PKC, a significant upstream regulator of NADPH oxidases that leads to their over-activation and subsequent increase ROS production in different retinal cells [85]. In brain, glutamate excitotoxic insults also increased the expression of the NOX2 isoform in reactive microglia cells [101]. Glutamate excitotoxicity and OS are two major players in DR. The relationship between diabetes and excitotoxicity was first addressed by Ambati et al. [102]. It has been reported that the levels of glutamate and other agents, gamma-aminobutyric acid (GABA) and vascular endothelial growth factor (VEGF), are increased in the vitreous of subjects with PDR. Diabetes increases glutamate levels in humans and rodents and alters glutamate uptake, thus interfering with retinal glutamate homeostasis, leading to retinal cell death [102,103,104]. Investigations that examined the effect of diabetes on glutamate metabolism, the glutamate transporter and glutamate receptors reported that diabetes produced enzymatic abnormalities in glutamate metabolism in retina [105], as well as dysfunction of the glutamate transporter [106] and alterations of ionotropic glutamate receptors [107,108]. All these dysfunctions have been observed in ESDR.

The relationship between OS and diabetes has also been addressed. It was reported that an increase in retinal superoxide production was observed in diabetic mice (2 and 8months) via various mechanisms that included NOX signaling. Apomycin (NOX inhibitor) suppressed the diabetes-induced increase in superoxide levels, specifically in the 8-month diabetic model, and reduced capillary degeneration [109]. Many investigations aimed in deciphering the inter-relationship amongst the increase in retinal glutamate levels, oxidative stress and nitric oxide in diabetes, and the effect and therapeutic potential of antioxidants in Diabetic Retinopathy [110,111,112].

The above-mentioned findings recommend for the first time that the above triad, diabetes, excitotoxicity and oxidative stress is interrelated. OS represents a putative link between diabetes and glutamate metabolic abnormalities in retina, which are further induced by excitotoxic insults.

## 5. Diabetic Retinopathy: NOX2 and NOX4

Expression of NOX1, NOX2, and NOX4 isoforms has been identified in different types of retinal cells, including macro- and microglia, pericytes, and endothelial cells [75,113,114]. NOX2 activity is also increased in diabetes [115].We have reported alterations in NOX2 and NOX4 expression in retina as early as two weeks after the induction of diabetes, increasing the nitration of protein tyrosine residues, and suggesting an increase in ROS levels and OS in ESDR [53,54]. Similar results were reported by investigators using other paradigms of ESDR, with different time scales (e.g., 15 or 20day or 4-week models), and in retinas of human subjects with diabetes [116,117]. The above-mentioned results and the reports of others [118] recommend that NOX2 and NOX4 isoforms are implicated in the induction of oxidative/nitrative stress and cell damage in diabetic retina.

### 5.1. NOX2 Inhibitors and Mechanisms of Action in the Neuroprotection of Early Diabetic Insults

Diabetes-induced excitotoxicity affects retinal neurons, leading to neurodegeneration and retinal cell death. NOX2 was found to be involved in AMPA-induced glutamate excitotoxicity [98] and DR [54].The role played by NOX2 activation on OS was reported initially in studies examining the induction of neuronal abnormalities by NOX, due to astroglial NF-κB mediating OS [119] and neuroprotection by the deletion of NOX2 isoform [120], in a model of ischemia/reperfusion injury. An increase in glutamate levels in retinas of diabetic animals was observed as an early diabetic insult, through the impairment of the excitatory amino acid transporter 1 (EAAT1), primarily responsible for the removal of excess glutamate from the extracellular space and expressed in Müller cells [54,121,122]. NOX2 blockade with the novel NOX2 inhibitor GLX7013170 (kindly provided by Glucox AB) depicted anti-apoptotic actions, partially protected the EAAT1 and reduced glutamate levels in retinas of diabetic non-treated animals, being primarily responsible for the removal of excess glutamate from the extracellular space [54]. Changes in EAAT1 expression and function are correlated with retinal neuronal loss due to excitotoxicity. Studies in brain have also identified NOX2 as a mediator for the excessive release of glutamate and the induction of excitotoxicity in pathological conditions such as stroke [123,124].

The above-mentioned findings allude to an interplay amongst the triad, NOX2, oxidative stress, and glutamate excitotoxicity that promotes neurodegeneration and neuroinflammation in DR.

### 5.2. NOX4-Neuronal Cell Death and Neuroprotection

It has been reported that NOX4 is over expressed in hypoxia and in high-glucose treated primary bovine retinal capillary endothelial cells in db/db mouse retina [55]. The novel NOX4 selective inhibitor GLX7013114 was reported to improve β-islet mitochondrial activity and survival in a model of short-term stress, suggesting that it may be a putative therapeutic for type 2 diabetes [125]. It also prevented human islet cell death [126]. The results of these studies recommended that NOX4 inhibition is an important strategy to pursue for the development of new therapeutics for diabetes and DR.

GLX7013114 (kindly provided by Glucox AB), administered as eye drops, blocked the early events of DR, with respect to nitrative stress [53], in agreement with its antioxidant and neuroprotective effects in the presence of AMPA [97].

An experimental intervention with GLX701 3114, employing a preventive (2week) and therapeutic (5week) paradigm of ESDR, led to neuroprotective, anti-inflammatory, and antivascular actions [53]. GLX7013114 blocked the NOX4 induced neuronal cell death, as depicted by the depletion of NOS-expressing amacrine cells in diabetic retina, as was previously reported for other therapeutic targets in ESDR STZ-DR models [50,51,127].Key pathological features of DR pertain to the loss of amacrine cells and the marked decrease in the thickness of the nerve fiber layer (NFL) [128]. NOX4 blockade was efficacious in blocking or reversing the diabetes-induced attenuation of NFL thickness, as early as two weeks, as well as the thickness or shrinkage of the areas corresponding to ganglion cell layers (GCLs) and inner plexiform layers (IPLs) [53]. Despite the reduction in NFL thickness and intensity, no reduction was observed in the thickness neither of the GCL nor in the number of RGCs between control and diabetic animals [52].

Changes in NFL thickness represent one of the earliest structural changes at the neuronal level of the diabetic retina, thus this will play an important role on the future development of DR [129]. Progressive loss of ganglion cells was reported after axotomy of the optic nerve [130].The existence of a large number of RGCs, with no detectable retrograde Fluoro-Gold staining, a marker for neuroanatomical tracing, was reported in a mouse model of glaucoma, suggesting that part of their axons was damaged, but RGCs remained functional and expressed various RGC genes. The authors suggested that despite the degeneration of their distal part, the proximal portion of the axons remained unaffected one-month post-insult, thus contributing to RGCs’ survival [131]. In the study by Dionysopoulou et al. [53], pattern electroretinography (pERG) analysis, a sensitive measure of RGC function, was performed depicting that NOX4 blockade with GLX7013114 protected the function of RGCs against the diabetic insult. Nerve fiber layer thinning in subjects with preclinical retinopathy [132], and subjects with diabetes with or without DR [133] indicated the existence of early structural alterations in the inner retina.

All the aforementioned findings suggest that GLX7013114 support the neuroprotective effects of GLX7013114 on specific retinal neurons (NOS-expressing amacrine cells) and RGC axons against the diabetic insults of ESDR. The treatment of the diabetes-induced NFL deficits in the ESDR will be beneficial towards the viability of RGCs and the postponement of the advanced stage of the disease.

### 5.3. NOX4 Blockade and Vascular Leakage

VEGF is responsible for vascular permeability and neovascularization in the diabetic retina. It has been reported that OS instigates a VEGF autocrine loop in Müller cells via a mechanism involving transcription factors responsible for the transcription, translation, and release of VEGF and its receptor, VEGFR2. According to these findings it was suggested that OS triggers the VEGF autocrine loop but also sustains it [134]. Therefore, it appears that VEGF may be involved in the early and late stages of DR. There are also other mechanisms besides OS that cause upregulation of VEGF during the development of DR, including hypoxia, AGEs, and pro-inflammatory cytokines [135]. VEGF is believed to have a dual role in DR, an initial neuroprotectant of retinal neurons against the hyperglycemia-induced OS and as a subsequent facilitator or enhancer of the progression of DR [136].

Diabetes induces an increase in VEGF levels in diabetic retina. NOX4 isoform is implicated in the diabetes-induced changes in VEGF levels in rat retina. Ocular topical administration of GLX7013114 reduced VEGF levels and vascular leakage, suggesting a protective role of this NOX4 inhibitor against diabetes-induced disruption of BRB integrity [53]. These actions of GLX7013114 support its performance as a therapeutic for ESDR. Increased VEGF levels and vascular leakage were reported in other ESDR models at one week [137], two weeks [138] and six months [139].

### 5.4. NOX2/NOX4 and Neuroinflammation

Microglia are the resident inflammatory cells in retina. Activated microglia increase the levels of pro-inflammatory cytokines and caspase-3 expression [140,141]. Zeng et al. [142] reported that the number of diabetes-induced activated microglia was increased in different layers of the diabetic retina, as early as the first month of diabetes. A paracrine mechanism was suggested to be involved in the regulation of microglia in the inner retina.

Retinal Müller cells (macroglia) also play a role in the inflammation process induced by diabetes, and are an important source of pro-inflammatory cytokines, including, TNF-α, IL-1β, IL-6, and VEGF. These cytokines induce impairments in glutamate metabolism, directly affecting retinal neurons, vascular cells, and the integrity of the BRB. VEGF mRNA expression has been reported in retinal neurons, glia, endothelial cells, pericytes, and Retinal Pigment Epithelium (RPE) [143,144]. Many reports have suggested that neighboring cells, such as neurons and vessels, may also release VEGF and increase its levels, aiding the progression of the disease to microangiopathy.

The NOX2 isoform has been mainly associated with vascular impairments that characterize DR. Diabetes increased activation of both micro- and macroglia results in significant production and release of pro-inflammatory cytokines. Pertaining to these actions, GLX701 3170, a NOX2 inhibitor, significantly reduced the over-activation of both micro- and macroglia in the ESDR model. Based on these results, NOX2 is involved in glial associated and neurodegenerative changes present in ESDR [54]. Similarly, NOX4 blockade by GLX701 3114, decreased micro- and macroglia reactivity and the expression of pro-inflammatory cytokines suggesting a putative immunomodulatory role [53].

## 6. NADPH Oxidases as a Link Between ESDR Major Pathologies

**Figure 3:**
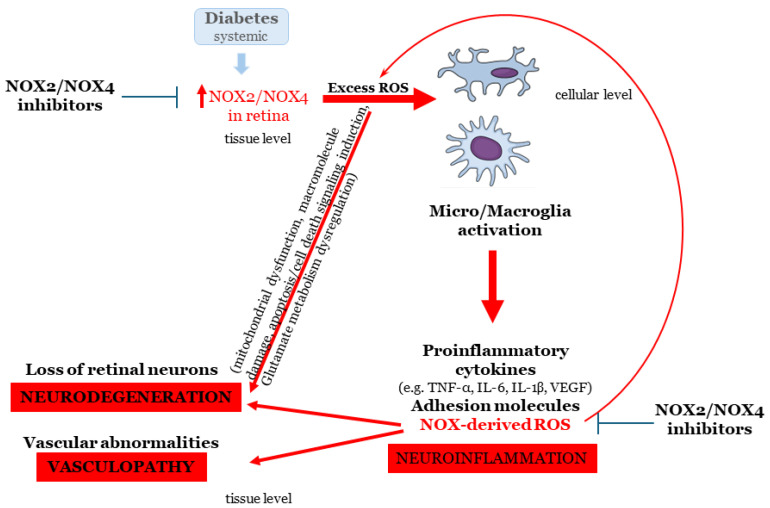
Schematic model of NADPH oxidase actions in ESDR.Diabetes-induced increase in NOX2 and NOX4 activation leads to the accumulation of ROS. This in turn initiates apoptosis, of neuronal and vascular cells, glial activation, and enhances inflammation (e.g., via NF-κB signaling pathway, VEGF expression). At a subsequent level, NOX-derived ROS produced by activated glial cells further deteriorates NVU by increasing neuroinflammation, vasculopathy and neurodegeneration. NADPH oxidase inhibition represents a promising therapeutic approach for the early-stage DR, thus ameliorating the progression of the disease.

All of the aforementioned preclinical data indicate that NADPH oxidases, NOX2, and NOX4 isoforms are involved in the early events of diabetes in retina. A putative mechanism of action is proposed, suggesting a dual role for NOX2 and NOX4. NOX-derived ROS act as regulators in the pathways that lead to the impairment of NVU at two levels. (1) Diabetes associated with the increase in the expression of NOX2 and NOX4 in the retina results in augmentation of ROS in the tissue. At this level, NOX-derived ROS trigger the activation of both micro- and microglia and activation of inflammatory pathways (e.g., NF-kB activation). They also initiate signaling pathways related to cell death/apoptosis, directly affecting retinal neurons and vascular cells. In addition, an up regulation in VEGF expression is also evident and contributes to vascular damage and the amplification of inflammation. (2) At the secondary level, NOX-derived ROS, produced as a result of glial activation, amplify the oxidative damage in the NVU, affecting neurons and vascular cells and initiating a loop of further induced activation of glial cells and neuroinflammation (Figure 3).

Based on this model, NADPH oxidase inhibitors can exhibit their protective actions on different levels: (a) attenuating the initial effect of oxidative stress on retinal neurons, glia and vascular cells and (b) preventing further oxidative damage in the tissue. Thus, they emerge as a promising target for further preclinical evaluation in ESDR.

## 7. Conclusions

The novel contribution of this review lies in the integration of emerging data suggesting a key role for NADPH oxidases as mediators of the oxidative stress effects on the diverse components of NVU in ESDR.

In this context, this review summarizes current knowledge on the diabetes-induced impairment of the NVU, through mechanisms involving ischemia, oxidative stress, and excitotoxicity. The complex interplay among this triad contributes to the three major pathologies of ESDR, namely neurodegeneration, vasculopathy, and neuroinflammation, with NADPH oxidases emerging as a putative mechanistic link across these pathologies (Figure 3).

Despite the advances in the understanding of DR development and progression, pharmaceuticals for the treatment of the pathologies of ESDR remain limited, rendering the urgent need for new treatments. NADPH oxidases have emerged as novel targets for the development of new therapeutics for the treatment of neurodegenerative disease (i.e., in brain and retina). GLX7013114 (NOX4) and GLX7013170 (NOX2) novel specific inhibitors are small molecules demonstrating broad protective effects when administered topically as eyedrops against the key pathological events of ESDR.

GLX7013114 has high affinity for NOX4, with no affinity for other NOX isoforms present in retina, and no scavenging or assay interference properties [53,60], fulfilling all the criteria that dictate the specificity of NOX inhibitors. A pharmacokinetic study substantiated that GLX7013114 reaches the retina when administered as eyedrops, justifying its efficacy and suggesting that a proper dose regimen of GLX7013114 may be beneficial for the treatment of ESDR and for delaying the development of ASDR and vision loss [53].

DR is also associated with other neurodegenerative diseases. Individuals with type 2 diabetes were reported to have a higher risk of developing neurodegenerative disease (e.g., Alzheimer’s disease) [145]. A Danish registry-based nationwide cohort study investigated whether diabetes and DR is a risk marker of AD. It was reported that individuals with DR had a 34% higher risk of incident AD, whereas individuals with diabetes without DR were less likely to develop AD compared to persons without diabetes [146].

The new knowledge evolved regarding the interplay between NOX2, oxidative stress and glutamate excitotoxicity in DR, and the molecular mechanisms responsible for the actions of NOX2 and NOX4 inhibitors in ESDR provide a better understanding for the development of new therapeutics. These will be beneficial not only for subjects with DR and other neurodegenerative diseases, but also individuals with other diabetic microvascular (diabetic cardiopathy, neuropathy, and kidney disease) and macrovascular (stroke, cardiovascular morbidity) complications, since DR is considered an independent predictor of all diabetic complications [147,148].

In closing, the findings of the preclinical investigation of NOX2 and NOX4 as therapeutics in the ESDR support their therapeutic potential. GLX7013170 and GLX7013114 inhibitors appear as lead molecules, something that must be validated clinically.

## Data Availability

No new data were created or analyzed in this study.
